# Evaluation of family history in individuals with heterozygous BRCA pathogenic variants diagnosed with breast or ovarian cancer in a single center in Italy

**DOI:** 10.1002/mgg3.2071

**Published:** 2022-10-28

**Authors:** Serena Negri, Elena De Ponti, Federica Paola Sina, Elena Sala, Cristina Dell'Oro, Gaia Roversi, Sara Lazzarin, Martina Delle Marchette, Alesssandra Inzoli, Claudia Toso, Simona Fumagalli, Maria Campanella, Joanne Kotsopoulos, Robert Fruscio

**Affiliations:** ^1^ Clinic of Obstetrics and Gynecology, Department of Medicine and Surgery University of Milan‐Bicocca Milan Italy; ^2^ Department of Physical Medicine ASST Monza, San Gerardo Hospital Monza Italy; ^3^ UOC Gynecologic Surgery ASST Monza, San Gerardo Hospital Monza Italy; ^4^ UO Medical Genetics ASST Monza, San Gerardo Hospital Monza Italy; ^5^ UOC Gestione Sanitaria delle Convenzioni ATS Brianza Lecco Italy; ^6^ aBRCAdabra ONLUS Milan Italy; ^7^ Women's College Research Institute Women's College Hospital Toronto Ontario Canada; ^8^ Dalla Lana School of Public Health University of Toronto Toronto Ontario Canada

**Keywords:** AIOM, *BRCA1/2* mutations, family history, genetic testing, NCCN

## Abstract

**Background:**

*BRCA1* and *BRCA2* gene mutations are responsible for 5% of breast cancer (BC) and 10–15% of ovarian cancer (EOC). The presence of a germline mutation and therefore the identification of subjects at high risk of developing cancer should ideally precede the onset of the disease, so that appropriate surveillance and risk‐reducing treatments can be proposed. In this study, we revisited the family history (FH) of women who tested positive for *BRCA* mutations after being diagnosed with BC or EOC.

**Methods:**

The National Comprehensive Cancer Network (NCCN) Clinical Practice Guidelines in Oncology (NCCN Guidelines®), and the Italian Association of Medical Oncology (AIOM) guidelines were applied to the FH of 157 women who were referred to San Gerardo Hospital for genetic counseling.

**Results:**

Almost 85% of women had an FH of *BRCA*‐related cancer. 63.7% and 52.2% of women could have undergone genetic testing according to NCCN and AIOM testing criteria (*p* < .05) before tumor diagnosis. An FH of EOC was the most frequent NCCN criterion, followed by BC diagnosed <45 years old. Sixty‐five percent of deceased women could have undergone genetic testing before developing cancer.

**Conclusions:**

FH is a powerful tool to identify high‐risk individuals eligible for genetic counseling and testing. Testing of healthy individuals should be considered when an appropriately affected family member is unavailable for testing.

## Graphical abstract

The presence of a germline BRCA1/2 mutation and the identification of subjects at high risk of developing cancer should ideally precede the onset of the disease, so that appropriate surveillance and risk‐reducing treatments can be proposed. In this study we overview the family history of women who tested positive for BRCA mutations after being diagnosed with breast or ovarian cancer. Almost respectively two third and half of patients met the NCCN and AIOM guidelines for genetic screening based on their family history alone. Therefore, in order to prevent BRCA related tumors, an adequate attention should be paid to family history.

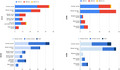

## 1 INTRODUCTION

Breast cancer (BC) has recently become the most commonly diagnosed cancer in the world, with about 2.3 million new cases in 2020. In women, it is also the first cause of cancer death (Sung et al., 2021).

Epithelial ovarian cancer (EOC) is the third more common gynecologic cancer but has the worst prognosis and the highest mortality rate (Bray et al., 2018; Coburn et al., 2017; Momenimovahed et al., 2019). It is also three times more lethal than BC (Momenimovahed et al., 2019; Yoneda et al., 2012). Most breast and ovarian cancers are sporadic, however, a germline pathogenic mutation in *BRCA1* or *BRCA2* genes is carried in approximately 5% of BC and 10–15% of EOC patients (Alsop et al., 2012; De Talhouet et al., 2020; Rebbeck et al., 2015). Women with *BRCA* mutations have an increased lifetime risk of developing BC and EOC, which can be up to 39% for EOC and 65% for BC depending on age and mutation type (Antoniou et al., 2003). These mutations are also linked to an increased risk for male breast, fallopian tube, primary peritoneal, pancreatic, prostate, and colon cancers, which are collectively known as *BRCA*‐related cancers (Lee et al., 2017; Mersch et al., 2015). The *BRCA1* and *BRCA2* gene mutations are inherited in an autosomal dominant pattern, which means that first‐degree relatives of a known carrier patient have a 50% chance of being carriers too. This is why investigating the family history (FH) of a patient is always crucial.

Many guidelines have been provided over time, both regionally and internationally, to identify individuals who could benefit from genetic counseling and testing. The criteria used to determine the eligibility of patients can differ from one to another, with some guidelines being more inclusive than others, and they all agree on some key points such as familial and personal histories of cancer (Forbes et al., 2019).

The National Comprehensive Cancer Network (NCCN) Clinical Practice Guidelines in Oncology (NCCN Guidelines®) (Daly et al., 2021) are among the best‐known and recently updated guidelines. They provide multiple sets of scenarios where testing could be clinically indicated, including a patient's relative with a known pathogenic/likely pathogenic variant in a cancer susceptibility gene, a characteristic personal history of cancer, or a suggestive FH. The Italian guidelines from the Italian Association of Medical Oncology (AIOM) are a landmark for Italian Oncology and are regularly updated (AIOM, n.d.). According to these guidelines, FH should only be investigated in association with a personal history of BC or prostate cancer, meaning that unaffected patients are not routinely supposed to be evaluated for testing, except in the case of two or more relatives with pancreatic cancer.

Testing an affected patient is usually considered the best option by geneticists and guidelines, as the chance to identify a pathogenic mutation is higher and the interpretation of a negative result is simpler. Moreover, the result of genetic testing can modify the treatment plan of mutation carriers. For example, a woman with EOC and a *BRCA* mutation can access targeted oncogenetic therapies like PARP inhibitors, in addition to the standard adjuvant chemotherapy, with a great benefit in terms of her oncological outcome (Banerjee et al., 2021).

However, identifying healthy subjects at high risk of developing cancer *before* the onset of the disease is one of the most important challenges in preventive medicine, because that means identifying the individuals that could benefit from appropriate surveillance and risk‐reducing treatments.

In this study, we revisited the FH of women who were referred to genetic counseling at San Gerardo Hospital and tested positive for *BRCA* mutations after being diagnosed with BC or EOC, to see if they already had a suggestive FH at the time of diagnosis and would have been therefore eligible for genetic counseling and *BRCA* testing before developing cancer.

## 2 SUBJECTS AND METHODS

### 2.1 Patients selection

Women who were referred to San Gerardo Hospital for genetic counseling after being diagnosed with BC or EOC and were found to be carriers of germline pathogenic mutations of *BRCA1/2* were considered for the analysis. Our Institution is a University Hospital (University of Milan‐Bicocca), with both a genetic division and an outpatient clinic dedicated to gynecologic follow‐up and treatment of women with a high genetic risk of developing malignancies. Only patients with complete information about their FH were included.

Clinical data collected included *BRCA* mutation, personal history of cancer, age at diagnosis of cancer and at genetic testing, FH of breast, ovarian, pancreatic or prostate cancer, age, and cause of death.

### 2.2 Testing criteria

“Testing criteria for high‐penetrance breast and/or ovarian cancer susceptibility genes” exposed on the National Comprehensive Cancer Network (NCCN) Clinical Practice Guidelines in Oncology (NCCN Guidelines®) for Genetic/Familial High‐Risk Assessment: Breast, Ovarian, and Pancreatic (Version 2.2021—January 6, 2021) (Rebbeck et al., 2015) were used as testing criteria. For pedigree evaluation, we adhered to the classification presented in the same guidelines (see Eval B). First‐degree relatives: parents, siblings, and children; second‐degree relatives: grandparents, aunts, uncles, nieces, nephews, grandchildren, and half‐siblings; third‐degree relatives: great‐grandparents, great‐aunts, great‐uncles, great‐grandchildren, first cousins, and half aunts and uncles.

As comparison, we used the Italian guidelines from AIOM, the Italian Association of Medical Oncology, “Raccomandazioni per l'implementazione del test *BRCA* predittivo e preventivo nei tumori della mammella, dell'ovaio, del pancreas e della prostata” (July 2021) (Alsop et al., 2012). Unaffected patients are not supposed to be evaluated for testing except in the rare case of at least two first‐degree relatives or three family members affected by pancreatic adenocarcinoma, therefore, FH criteria alone, originally meant to be associated with a personal history of BC ≤50 years, were applied: EOC at any age, BC diagnosed at age <50 years, bilateral BC diagnosed at any age, male BC diagnosed at any age, locally advanced or metastatic pancreatic cancer, and metastatic prostate cancer. According to these criteria, the only significant relatives are parents, siblings, and children; paternal grandparents, paternal aunts, and uncles are taken into account only if affected by BC or EOC.

### 2.3 Statistical analysis

Absolute frequencies and percentages were used to describe the categorical patients' population. Continuous findings are summarized by median value and range. Chi‐squared test and Fisher's exact test were used to estimate the *p* values for associations between FH criteria and patients' characteristics. A *p* value of < .05 was considered the threshold of statistical significance.

## 3 RESULTS

**FIGURE 1:**
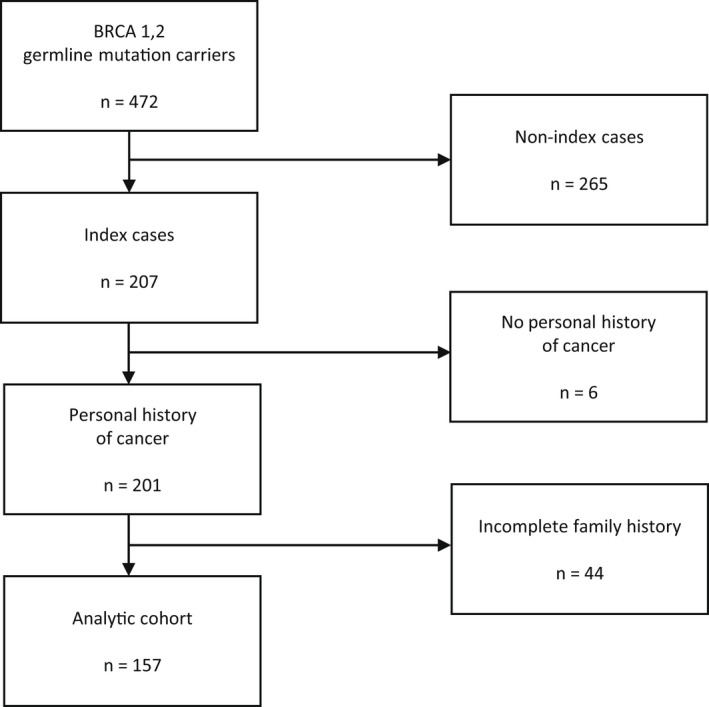
Patient selection flowchart

Among 472 women carrying pathogenic *BRCA1/2* germline mutations diagnosed between April 2010 and April 2020 followed in our outpatient clinic, we selected individuals that were considered index cases (*n*: 207). About 201 women had a history of cancer and were selected for this analysis; however, 44 patients were excluded due to an unknown FH. Therefore, a final population of 157 women was considered (Figure 1).

Patient characteristics are listed in Table 1. The median age at genetic testing was 52 years (range 22–77 years), whereas the median age at diagnosis of cancer was 46 years (range 22–73 years). Eighty‐four women (53.5%) had BC, 55 (35%) had EOC, and 18 (11.5%) had both. Almost 85% of women (133 patients) had an FH of *BRCA*‐related cancer, involving a first‐degree relative in 67.7% of the cases.

**TABLE 1:** Patients characteristics (*n* = 157)

Characteristics	No. patients (%)
Mutation	
*BRCA1*	87 (55,41)
*BRCA2*	69 (43,95)
*BRCA1 + 2*	1 (0,64)
Age at genetic testing (years)	
*Median age: 52 years (range 22–77)*	
<35	12 (7,64)
35–45	42 (26,75)
45–55	47 (29,94)
>55	56 (35,67)
Cancer diagnosed	
Breast cancer	84 (53,5)
Ovarian cancer	55 (35,0)
Both	18 (11,5)
Age at cancer diagnosis (years)	
*Median age: 46 years (range 22–73)*	
<35	17 (10,83)
35–45	57 (36,31)
45–55	51 (32,48)
Family history	
No	24 (15.29)
Yes	133 (84,71)
I grade relative	90 (67,7)

One hundred women (63.6%) could have undergone genetic testing according to NCCN testing criteria before developing cancer themselves. In particular, 45 women had an FH of EOC; 33 had an FH of BC diagnosed at age ≤45 years; 14 had an FH of BC diagnosed at age 46–50 years with a second BC diagnosed at any age or with ≥1 close blood relative with breast, ovarian, pancreatic, or high‐grade (Gleason score ≥7) or intraductal prostate cancer at any age; 10 women had an FH of BC diagnosed at any age with ≥1 close blood relative with BC at age ≤ 50 years or ovarian, pancreatic, or metastatic or intraductal prostate cancer at any age; 5 women had an FH of male BC diagnosed at any age; and 15 had an FH of exocrine pancreatic cancer at any age. Twenty women fell into more than one criterion for testing. For each of these groups of women, we analyzed the degree of kinship of the affected relative they had, the type of mutation they carried, and the type of cancer they developed (Table 2).

**TABLE 2:** Degree of kinship, type of mutation, and personal history for each NCCN criterion

NCCN testing criteria	Degree of kinship		Mutation type			Personal history of cancer		
	I grade	II grade	*BRCA1*	*BRCA2*	*BRCA1 + 2*	BC	EOC	Both
Family history of breast cancer diagnosed at age ≤ 45 years	18	15	21	12	0	21	11	1
Family history of breast cancer diagnosed at age 46–50 years with *A second breast cancer diagnosed at any age*; ≥*1 close blood relative with breast, ovarian, pancreatic, or high‐grade/intraductal prostate cancer at any age*	7	7	6	8	0	10	3	1
Family history of breast cancer diagnosed at any age with ≥*1 close blood relative with breast cancer at age ≤50 years or ovarian, pancreatic, or metastatic/intraductal prostate cancer at any age;s = D* great‐grandparents] ≥*3 total diagnoses of breast cancer in patient and/or close blood relatives*	6	4	6	4	0	7	3	0
Family history of male breast cancer at any age	4	1	1	4	0	2	3	0
Family history of epithelial ovarian cancer at any age	28	17	29	15	1	18	22	5
Family history of exocrine pancreatic cancer at any age	8	7	8	6	1	4	9	2

Eighty‐two women (52.2%) would have been detected as eligible for *BRCA* testing by applying AIOM FH criteria. In particular, 32 women had an FH of EOC; 37 women had an FH of BC diagnosed at age ≤50 years; 3 women had an FH of bilateral BC; 5 women had an FH of male BC diagnosed at any age; and 8 women had an FH of locally advanced or metastatic pancreatic cancer. Two women fell into more than one criterion for testing. The type of mutation they carried and the type of cancer they developed are shown in Table 3.

**TABLE 3:** Type of mutation and personal history for each AIOM criterion

AIOM testing criteria	Mutation type			Personal history of cancer		
	*BRCA1*	*BRCA2*	*BRCA1 + 2*	BC	EOC	Both
Family history of breast cancer diagnosed at age < 50 years	20	17	0	23	12	2
Family history of epithelial ovarian cancer at any age	19	12	1	12	16	4
Family history of bilateral breast cancer diagnosed at any age	2	1	0	3	0	0
Family history of male breast cancer at any age	1	4	0	2	3	0
Family history of locally advanced or metastatic pancreatic cancer	4	4	0	2	5	1

**FIGURE 2:**
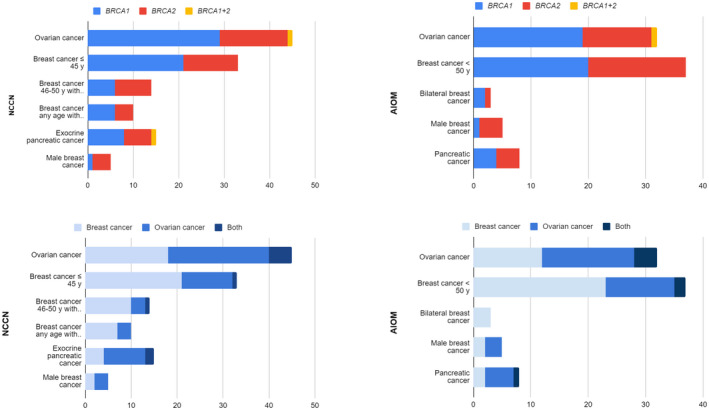
association between FH criteria according to NCCN or AIOM guidelines and the type of mutations (a) and with the type of cancer (b)

The association between each FH criterion according to NCCN or AIOM guidelines and the type of mutations are shown in Figure 2a, whereas the association between the type of cancer is shown in Figure 2b.

Twenty‐one women (13.4%) would have been detected by NCCN criteria and not by AIOM ones, due to just an FH of pancreatic cancer in a second‐degree relative (3 women, 14.3%), an FH of BC over or at 50 years in association with other affected relatives (11 women, 52.4%), an FH of tubal cancer (1 woman, 4.7%), and the inclusion of the maternal aunt among the significant family members (6 women, 28.6%).

On the other hand, 3 women (0.02%) would have been detected applying AIOM FH criteria but not the NCCN criteria, due to an FH of BC diagnosed between 46 and 50 years with no need for further affected family members (2 women, 66.6%) and to an FH of bilateral BC at any age (1 woman, 33.3%).

Fifty‐four women (34,4%) would have not been detected by neither NCCN nor AIOM criteria. Among them, 30 (55.5%) had an FH of *BRCA*‐related cancer which did not fit into any guidelines' criteria, with third‐ and fourth‐grade relatives as the only family members affected in 20% of cases.

Seventeen women (10.8%) died from tumor progression. The clinical characteristics of women are listed in Table 4. The median age at death was 54 years (range 25–77 years), whereas the median age at diagnosis of cancer was 50 years (range 22–64 years). Thirteen women (76.5%) had EOC, 2 (11.75%) had BC, and 2 (11.75%) had both. About 82.3% of women had an FH of cancer, involving a first‐degree relative in 71.4% of cases. Eleven patients (64.7%) would have been detected by applying NCCN criteria and 4 women (36.3%) fulfilled more than one criterion: 10 women had an FH of EOC, 2 women had an FH of BC diagnosed at age ≤45 years; 1 woman had an FH of BC diagnosed at age 46–50 years with ≥1 close blood relative with breast, ovarian, pancreatic, or high‐grade (Gleason score ≥7) or intraductal prostate cancer at any age; 1 woman had an FH of BC diagnosed at any age with ≥1 close blood relative with BC at age ≤50 years or ovarian, pancreatic, or metastatic or intraductal prostate cancer at any age; 1 woman had an FH of exocrine pancreatic cancer at any age. Nine women (53%) would have been detected by applying AIOM FH criteria: seven women had an FH of EOC and two women had an FH of BC diagnosed at age ≤50 years.

**TABLE 4:** Deceased patients’ characteristics

Characteristics	No. patients (%)
Mutation	
*BRCA1*	10 (58.8)
*BRCA2*	6 (35.3)
*BRCA 1 + 2*	1 (5.9)
Cancer diagnosed	
Breast cancer	2 (11.7)
Ovarian cancer	13 (76.6)
Both	2 (11.7)
*Median age at diagnosis: 50 years (range 22–64)*	
*Median age at death: 54 years (range 25–77)*	
Family history	
No	3 (17.6)
Yes	14 (82.4)
Meeting criteria	
NCCN	11 (64.7)
Epithelial ovarian cancer at any age	10 (90,9)
Breast cancer diagnosed ≤45 years	2 (18.2)
Breast cancer diagnosed at age 46–50 years with	1 (9.09)
Breast cancer diagnosed at any age with	1 (9.09)
Exocrine pancreatic cancer at any age	1 (9.09)
AIOM	9 (53)
Epithelial ovarian cancer at any age	7 (77.8)
Breast cancer diagnosed at age < 50 y	2 (22.2)

## 4 DISCUSSION

In 1990, when Dr. Marie‐Claire King demonstrated that a gene located on chromosome 17 was linked to early onset familial BC, she concluded a hunt lasting more than 15 years. Her work got started by the observation that, in some families, the frequency of BC was much higher than expected, and also that the age of onset of the disease was lower compared with the rest of the female population. In terms of preventive medicine, the knowledge about the mutational status of *BRCA1* and *BRCA2* has become of utmost importance, especially in women, as it allows the planning of an appropriate surveillance program and, when indicated, of risk‐reducing therapies or surgical interventions. Moreover, it also enables rational and conscious decisions about lifestyle, family planning, and contraception. Despite the great number of clear advantages deriving from the knowledge of the presence of mutations in cancer susceptibility genes, there is a generalized massive underutilization of genetic testing. Even if evidence highlights the cost‐effectiveness of a population‐based approach for genetic testing (Manchanda et al., 2018), current guidelines generally recommend the use of algorithms (e.g., BOADICEA, *BRCA*PRO) to detect individuals with a risk above the threshold for testing (10%). The guidelines for referring women to genetic counseling mainly rely on the FH, but, even in countries with an advanced healthcare system as the USA, no more than 20% of eligible women access and undergo genetic testing (Childers et al., 2017). This is true also in our population, as less than 3% of women (6/207) considered index cases underwent genetic testing without a previous diagnosis of cancer. This clearly represents a huge missed opportunity and, in the majority of cases, we identify pathogenic mutation carriers only after one of their relatives gets cancer. Since this diagnosis could have been potentially prevented, it represents a failure and must be absolutely avoided.

In our population, almost 85% of the patients had at least one relative affected by a type of cancer known to be associated with *BRCA* mutations (BC, EOC, exocrine pancreatic cancer, or prostate cancer). In most cases, a first‐degree relative was involved, being the mother more frequently. Applying the NCCN Clinical Practice Guidelines and the AIOM Guidelines to each of our patient's FH, we found out that 65.6% of women (103 patients) met at least one of those criteria and were therefore eligible for testing before developing cancer.

A significantly lower proportion of patients (*p*: .026) could have been eligible for genetic testing according to AIOM criteria compared with NCCN criteria. In fact, 52.2% of patients (82 women) in our study could have been eligible for testing according to the AIOM criteria, as opposed to 63.7% of the patients (100 women) identified by applying NCCN criteria. The main difference lies in a more comprehensive study of the pedigree, which includes all first‐ and second‐degree relatives of the patients and leads to the possibility of testing: in case of an FH of BC over or at 50 years if there are other affected relatives, if the only affected family member is on the maternal side of the family, and if a second‐degree relative is affected by pancreatic or prostate cancer.

The single most frequent NCCN criterion was an FH of EOC (45 women), followed by an FH of BC diagnosed under 45 years (33 women). As for the AIOM criteria, the most frequent one was an FH of BC diagnosed under 50 years (37 women) followed by an FH of EOC (32 women). This reinforces the strong and well‐known association between *BRCA* mutations and ovarian and BC, especially when they occur at a young age.

The association between *BRCA* mutations and exocrine pancreatic cancer has been more recently described. Approximately 0.7%–5.7% and 0.3%–2.3% of unselected cases of pancreatic adenocarcinoma are linked to pathogenic mutations, respectively, in *BRCA1* and *BRCA2* genes (Rosen et al., 2021), making them the most common cause of familial pancreatic cancer (Pilarski, 2019). The prevalence of mutations among these patients and the poor prognosis of this type of cancer has led the NCCN Guidelines to offer genetic counseling and testing to all patients with a personal or FH of pancreatic adenocarcinoma (Pilarski, 2019). Pancreatic cancer has been included as a testing criterion in the AIOM guidelines only since May 2021, when an update has been published. What is noteworthy is that it is the only kind of FH that does not have to be necessarily associated with a personal history of cancer to lead to genetic counseling, meaning that it is the only scenario where an unaffected patient can be tested according to the AIOM guidelines. Fifteen women (9.5%) in our study could have undergone genetic testing for an FH of exocrine pancreatic cancer in a first‐ or second‐degree relative. Interestingly, among these patients, a significantly more frequent personal history of EOC (*p*: .027) was observed. There was no difference in the prevalence of *BRCA1* or *BRCA2* mutations.

The association between an FH of EOC and a personal history of EOC did not reach statistical significance (*p*: .076). The role of an FH of EOC as a predictor of EOC risk is already demonstrated, especially among *BRCA1* mutation carriers (Texeira et al., 2018); this is highly relevant considering that EOC is the third most common gynecologic cancer and has the worst prognosis and the highest mortality rate (Bray et al., 2018; Coburn et al., 2017; Momenimovahed et al., 2019).

Among the 17 deceased patients in our study, 15 (88.2%) died of EOC. Eleven women (65%) could have undergone genetic counseling and testing before developing cancer according to the NCCN or the AIOM criteria and the most frequent criterion was an FH of EOC (NCCN: 10 patients; AIOM: 7 patients).

Our results show that in a population of women who were discovered to be *BRCA* mutation carriers after a diagnosis of cancer, their FH alone would have allowed physicians to propose genetic counseling and testing when they were still healthy in most cases. Missing this opportunity has deprived them of effective primary prevention and can be considered a failure in cancer prevention in general.

*BRCA* mutations can be observed in patients without an FH of cancer and are therefore detected after a diagnosis of cancer (Grindedal et al., 2017; Singer et al., 2019): this eventuality is of course nonpredictable and could be prevented only by a population‐based screening (Daly et al., 2021; Gabai‐Kapara et al., 2014). Since the *BRCA* gene test is not yet routinely performed on people at average risk of breast and ovarian cancers, our best practice as physicians is to identify women at high risk when they are still healthy. In order to do so, the general practitioner has a crucial role, as she/he has the chance and should always accurately investigate the FH of patients to select potential candidates for genetic counseling.

Many surveys both in Europe and in the U.S. have been conducted to assess attitude and knowledge regarding predictive genetic testing for cancer among physicians (Bellcross et al., 2011; Marzuillo et al., 2013, 2014; Nippert et al., 2011), pointing out the strong need for targeted educational programs. These could be particularly useful to improve awareness of the importance of EOC personal/family history since it seems to trigger a referral to genetic counseling less than a personal/family history of BC (Febbraro et al., 2015; Powell et al., 2013).

Taken together, our data suggest that the identification of women with a high risk of developing cancer is possible, and FH could help identify subjects that should be tested. In this regard, AIOM guidelines could probably benefit from the addition of FH alone (without a personal history of malignancies) among the criteria to select subjects for genetic testing. Since universal testing is unrealistic in our Country, at least at the present, a careful examination of FH seems to provide the opportunity to prevent a significant number of cancer cases.

One of the limitations of this study consists of the reduced size of the study population, which did not allow the analysis of each criterion's association with *BRCA* mutations or a personal history of cancer. Moreover, we could not recollect the histology and the stage of tumors of most of our patients' family members: we may have missed some triple‐negative breast cancers (a criterion for testing in itself if diagnosed under or at 60 years old) and high‐grade or intraductal prostate cancers. *BRCA*PRO was not calculated, as a personal history of cancer is a factor in calculating the percentage and was not removable. Finally, we cannot estimate the sensibility and specificity of the NCCN and AIOM criteria in the general population, as we do not know how many women without *BRCA* mutations have the same FH.

## 5 CONCLUSIONS

Even if far from being perfect, FH is a powerful tool to identify high‐risk individuals eligible for genetic counseling and testing, and the application of specific sets of criteria, which should be included in guidelines, can be useful to guide this decision. Testing of healthy individuals should be considered when an appropriately affected family member is unavailable for testing. An FH of EOC should suggest the possibility of the presence among the family of a *BRCA* mutation: all patients with a first‐ or second‐degree relative affected by EOC should be addressed to genetic counseling.

## AUTHOR CONTRIBUTIONS

Serena Negri, Elena De Ponti, and Robert Fruscio conceived and designed the work that led to the submission. Serena Negri, Federica Paola Sina, Cristina Dell'Oro, Sara Lazzarin, Martina Delle Marchette, and Simona Fumagalli acquired data. Elena De Ponti was responsible for statistical analysis. Serena Negri, Elena De Ponti, Elena Sala, Gaia Roversi, Claudia Toso, Alesssandra Inzoli, Maria Campanella, Joanne Kotsopoulos, and Robert Fruscio played an important role in interpreting the results. Serena Negri, Joanne Kotsopoulos, and Robert Fruscio Drafted and revised the manuscript. All the authors approved the final version. Robert Fruscio agreed to be accountable for all aspects of the work in ensuring that questions related to the accuracy or integrity of any part of the work are appropriately investigated and resolved.

## FUNDING INFORMATION

The authors received no specific funding for this work. Joanne Kotsopoulos is a recipient of Tier II Canada Research Chair.

## CONFLICT OF INTEREST

All the authors disclose any possible conflict of interest. None have received any support, for the work, in the form of grants and/or equipment and drugs.

## ETHICS STATEMENT

The analysis has been approved by the Ethical Committee “Brianza” on July 30, 2018 (#2876).

## Data Availability

The data that support the findings of this study are available from the corresponding author upon reasonable request.
